# Introducing a new exchange functional by altering the electron density’s ionization dependency in density functional theory

**DOI:** 10.1038/s41598-024-53341-4

**Published:** 2024-02-08

**Authors:** E. Rahmatpour, A. Esmaeili

**Affiliations:** Department of Physics, Urmia University, Urmia, Iran

**Keywords:** Physical chemistry, Materials science, Physics

## Abstract

Each of the exchange–correlation functionals in the density functional theory has been customized to particular systems or elements and has unique advantages and disadvantages. In one of the most recent research on exchange–correlation functionals, Chachiyo et al. present a relationship for exchange–correlation functional by assuming the simplest form of electron density. Their utilized electron density causes a systematic inaccuracy in the energy of the molecules since it does not fully account for the variation of the ionization energy for different atoms. We offer a novel relationship for exchange functional that improves the precision of the energy calculations for molecules by using the basic assumptions of the Chachiyo approach and correcting the electron density. Our density is directly related to the atom’s ionization energy. Our suggested functional was implemented for 56 molecules composed of atoms from the first, second, and third rows of the periodic table using Siam Quantum package. We discussed about the role of our functional on the reducing the computation error of dipole moment along with total, bonding and zero point energies. We also increased the portion of core electrons to improve the accuracy of the results.

## Introduction

**Figure 1:**
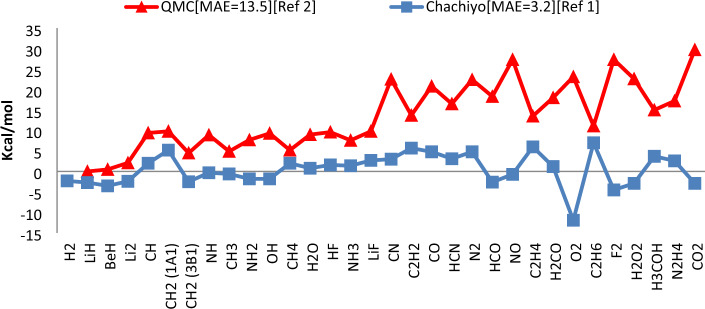
Total energy prediction error in terms of kilojoules/mol obtained from quantum Monte Carlo method and Chachiyo model for molecules containing atoms of the first and second rows of the periodic table^1^.

Quantum Monte Carlo (QMC) is employed to obtain one of the most precise estimates for the total energy of atoms in the framework of many particle systems^1^. As shown in Fig. 1, the mean absolute error (MAE) of QMC for molecules made from the first row of the periodic table and other rows, respectively, is 13.5 and 23.7 kcal/mol. Since QMC computations are very time-consuming, other approaches are currently being developed. Density functional theory (DFT) is one of them. It uses the electron correlation, kinetic and exchange energies, nuclear-electron interaction, and classical electron–electron Coulomb repulsion to determine the energy of a system. Since the correlation term is not taken into consideration in Hartree–Fock (HF) theory, the exchange energy determined precisely in HF cannot be employed in DFT^2^. Exchange energy can be calculated using a variety of approaches. These approaches depend on the electron density (n) as well as its gradients. Determining an exact exchange functional specially in the intermediate region, where electron density decays outside molecules between slow and rapid variation limit, is still under discussion. The primary aim of this work is to obtain exchange energy values that lead to results that are more accurate than QMC in all regions.

As predicted by the Thomas–Fermi model, the exchange energy enhancement in a system with slowly varying density is proportional to $(1+\mu {S}^{2})$, where S is a function of the reduced density gradient and is equal to $S=\left|\nabla n\left(r\right)\right|/[2{\left(3{\pi }^{2}\right)}^\frac{1}{3}{\left(n\right)}^\frac{4}{3}]$. According to the Kleinman model and taking into account an alternative external potential with a long wavelength as a perturbation, the $\mu$ coefficient has a constant value of 8/21^3^. In order to achieve more precise results for the total energy, Chachiyo et al. added the Bragg scattering condition for Fermi electrons and obtained a greater value for $\mu$^1^. Then, using the assumption that the electron density decays exponentially as $n\left(r\right)\to N{e}^{-ar}$, they offered a novel relationship for the exchange energy that led to accurate results for the total energy, particularly for a system with intermediate varying density^1^. As can be seen in Fig. 1, the error value of Chachiyo model (CM) for computing the total energy of molecules is acceptable and better than QMC.

The work by Chachiyo et al. actually misses a key aspect because the ionization energy is different for various atoms and the same relation for electron density should not be used for all of them. In this work, we utilized the density of electrons as $n\left(r\right)\to A{r}^{2\beta }{e}^{-2{\left(2I\right)}^\frac{1}{2}r}$ that clearly depends on the atom’s ionization^4^. We analytically derive a new relation for the exchange functional that is more accurate than previous attempts and works well in all slow, rapid, and intermediate varying density regions. To verify the accuracy of our results for different kinds of structures, we employed a set of reference data^5^.

## Theoretical method

An interacting many-particle system is described by the DFT exclusively in terms of its density and the system energy is stated as^6–8^:

1 $$E\left(n\left(\overrightarrow{r}\right)\right)=T\left(n\left(\overrightarrow{r}\right)\right)+U\left(n\left(\overrightarrow{r}\right)\right)+\int n\left(\overrightarrow{r}\right){V}_{ion}\left(\overrightarrow{r}\right){d}^{3}r,$$

2 $$E\left(n\left(\overrightarrow{r}\right)\right)={\text{F}}\left(n\left(\overrightarrow{r}\right)\right)+\int n\left(\overrightarrow{r}\right){V}_{ion}\left(\overrightarrow{r}\right){d}^{3}r .$$

F and V_ion_, respectively, stand for universal function and external potential in Eq. (2). The charge density contains all the information related to the system’s ground state^9^.

3 $$n\left(\overrightarrow{r}\right)\equiv \sum_{i}{n}_{i}{\left|\phi \left(\overrightarrow{r}\right)\right|}^{2},$$

And the density is calculated using the variational method as follows:

4 $$\frac{\delta }{\delta n\left(\overrightarrow{r}\right)}\left[E\left[n\left(\overrightarrow{r}\right)\right]-\mu \int {d}^{3}rn\left(\overrightarrow{r}\right)\right]=0 .$$

Equation (2) may also be rewritten as follows:

5 $$F\left[n\left(\overrightarrow{r}\right)\right]={{\text{T}}}_{s}\left[n\left(\overrightarrow{r}\right)\right]+\frac{1}{2}\int n\left(\overrightarrow{r}\right){V}_{H}\left[n\left(\overrightarrow{r}\right)\right]{d}^{3}r+{E}_{xc}\left[n\left(\overrightarrow{r}\right)\right],$$

where ${E}_{xc}^{0}\left[n\right]$ is the exchange correlation (XC) energy, which is defined by the following relationship:

6 $${E}_{xc}\left[n\right]=\int n\left(\overrightarrow{r}\right){\varepsilon }_{xc}\left[n\left(\overrightarrow{r}\right)\right]{d}^{3}r ,$$

where ${\varepsilon }_{xc}\left[n\left(\overrightarrow{r}\right)\right]$ represent exchange energy per electron. Gradient corrections are required in Eq. (6) to take into account the long-range gradient effects. The generalized gradient approximation (GGA) is the model that accomplishes this as:

7 $${E}_{{\text{xc}}}^{{\text{GGA}}}\left[n\left(r\right)\right]=\int n\left(r\right){\varepsilon }_{{\text{x}}}^{{\text{LSDA}}}\left(n\left(r\right)\right){F}_{{\text{xc}}}^{{\text{GGA}}}\left(n\left(r\right),\nabla n\left(r\right)\right)d\tau .$$

As mentioned earlier, the density in Eq. (7) is employed as follows in our model^4^:

8 $$n\left(r\right)\to A{r}^{2\beta }{e}^{-2r{\left(2I\right)}^\frac{1}{2}} ,$$

where $\beta =\left(\frac{1}{\sqrt{2I}}\right)-1$. For this density, $\left|\nabla n\left(r\right)\right|$ is obtained as:

9 $$\left|\nabla n\left(r\right)\right|=A{e}^{-2r\sqrt{2I}}{r}^{\sqrt{\frac{2}{I}} -3}\left(2-\sqrt{\frac{2}{I}} +2r\sqrt{2I}\right) .$$

In Eq. (8), I stands for the ionization energy and A is a constant. Equations (8) and (9) result:

10 $$\frac{\left|\nabla n\left(r\right)\right|}{{(n\left(r\right))}^\frac{4}{3}}=\frac{A{e}^{-2r\sqrt{2I}}{r}^{\sqrt{\frac{2}{I}} -3}\left(2-\sqrt{\frac{2}{I}} +2r\sqrt{2I}\right)}{{A}^\frac{4}{3}{e}^{-\frac{8}{3}r\sqrt{2I}}{r}^{\frac{4}{3}\sqrt{\frac{2}{I}} -\frac{8}{3}}}={A}^{\frac{-1}{3}}{e}^{\frac{2}{3}r\sqrt{2I}}{r}^{\frac{-1}{3}\sqrt{\frac{2}{I}} -\frac{1}{3}}\left(2-\sqrt{\frac{2}{I}} +2r\sqrt{2I}\right).$$

For a homogeneous electron gas, the Dirac exchange energy per electron, or ${\varepsilon }_{x}$, equals^10^:

11 $${\varepsilon }_{x}=\frac{-3}{4}{\left(\frac{3n}{\pi }\right)}^{1/3}.$$

The density determined by Eq. (8) yields the value of ${\varepsilon }_{x}$ as follows:

12 $${\varepsilon }_{x}=\frac{-3}{4}{\left(\frac{3}{\pi }A{r}^{2\beta }{e}^{-2r{\left(2I\right)}^\frac{1}{2}}\right)}^{1/3}=-\frac{3}{4}{\left(\frac{3}{\pi }\right)}^\frac{1}{3}{A}^\frac{1}{3}{e}^{\frac{-2}{3}r\sqrt{2I}}{r}^{\frac{1}{3}\sqrt{\frac{2}{I}} -\frac{2}{3}} .$$

Additionally, by using following equation for *S*^10^:

13 $$S=\frac{\left|\nabla n\left(r\right)\right|}{2{\left(3{\pi }^{2}\right)}^\frac{1}{3}{\left(n\right)}^\frac{4}{3}}.$$

As the reduced gradient parameter in the primary E_x_ equation, the exchange energy will take the form:

14 $${E}_{x}\left[n\right]=\int n{\varepsilon }_{x}F\left(S\right){d}^{3}r.$$

Therefore, S can be expressed as follows using Eq. (10):

15 $$S=\frac{1}{2{\left(3{\pi }^{2}\right)}^{1/3}}{A}^{\frac{-1}{3}}{e}^{\frac{2}{3}r\sqrt{2I}}{r}^{\frac{-1}{3}\sqrt{\frac{2}{I}} -\frac{1}{3}}\left(2-\sqrt{\frac{2}{I}} +2r\sqrt{2I}\right).$$

In the limit of $r\to \infty$,

16 $$S\cong \frac{2\sqrt{2I}}{2{\left(3{\pi }^{2}\right)}^\frac{1}{3}}{A}^{\frac{-1}{3}}{e}^{\frac{2}{3}r\sqrt{2I}}{r}^{\frac{-1}{3}\sqrt{\frac{2}{I}} +\frac{2}{3}}.$$

Now, using Eq. (12) and this equation for S, we can derive the following equation for ${\varepsilon }_{x}$:

17 $${\varepsilon }_{x}=-\frac{3\sqrt{2I}}{4\pi S}.$$

At the asymptotic limit of $S\to \infty$, the exchange energy density should behave as^10^:

18 $${\varepsilon }_{x}F\left(S\right)\to -\frac{1}{2r}\hspace{0.17em}\hspace{0.17em}.$$

Since the value of the optimum coefficient should be equal to 1 at S = 0 limit, F_x_(S) is expressed as follows:

19 $${F}_{x}\left(S\right)=\frac{2\pi S}{3r\sqrt{2I}}.$$

The relationship between r and S can be identified through Eq. (16). Assuming:

20 $$\alpha =\frac{{3}^{\frac{-1}{\sqrt{\frac{2}{I}} -2}}\sqrt{I}{\pi }^{\frac{-2}{\sqrt{\frac{2}{I}} -2}}{\left(\frac{{2}^{\frac{1}{2}-\frac{1}{\sqrt{2I}}}{A}^\frac{1}{3}}{\sqrt{I}}\right)}^{\frac{-3}{\sqrt{\frac{2}{I}} -2}}}{\sqrt{\frac{2}{I}} }-2.$$

And using *W* as the Lambert function, we obtain:

21 $$r=\left(\frac{\sqrt{2}}{2\sqrt{I}}-\frac{1}{2I}\right)W\left(-\alpha {S}^{\frac{-3}{\sqrt{\frac{2}{I}} -2}}\right)\hspace{0.17em}\hspace{0.17em}\hspace{0.17em}.$$

In this case, F_x_(S) is resulted as:

22 $${F}_{x}\left(S\right)=\frac{4\pi S}{3\left(2-\sqrt{\frac{2}{I}} \right)W\left(-\alpha {S}^{\frac{-3}{\sqrt{\frac{2}{I}} -2}}\right)}.$$

Equation (22) is obtained in the asymptotic range S → ∞. A weight function is required to extend the application of this formula to the intermediate range between S → ∞ and S → 0 limits. ${F}_{x}\left(S\right)$ should not diverge to infinity in the limit *s* → 0, thus we modify its functional as follows:

23 $${F}_{x}\left(S\right)=\frac{4\pi S}{3\left(2-\sqrt{\frac{2}{I}} \right)\left(W\left(-\alpha {S}^{\frac{-3}{\sqrt{\frac{2}{I}} -2}}\right)+1\right)}.$$

The weight function is then introduced as $\upomega \left(S\right)=\frac{1}{dS+1}$ where d is a constant that controls the weight function's speed from the ranges between low variation range to the asymptotic limit. Since the value of the functional at S = 0 must be equal to 1, we rewrite the weight function as follows:

24 $$F\left(S\right)=1.\upomega \left(S\right)+\frac{4\pi S}{3\left(2-\sqrt{\frac{2}{I}} \right)\left(W\left(-\alpha {S}^{\frac{-3}{\sqrt{\frac{2}{I}} -2}}\right)+1\right)}\left(1-\upomega \left(S\right)\right) =\frac{4\pi d{S}^{2}+3({d}^{2}{S}^{2}+dS+1)\left(2-\sqrt{\frac{2}{I}} \right)\left(W\left(-\alpha {S}^{\frac{-3}{\sqrt{\frac{2}{I}} -2}}\right)+1\right)}{3\left(dS+1\right)\left(2-\sqrt{\frac{2}{I}} \right)\left(W\left(-\alpha {S}^{\frac{-3}{\sqrt{\frac{2}{I}} -2}}\right)+1\right)} .$$

We derive the following series for the functional by using Taylor expansion up to the second order around the zero point:

25 $$S\ll 1, F\left(S\right)\approx 1+\left({d}^{2}+\frac{4\pi d}{3\left(2-\sqrt{\frac{2}{I}} \right)}\right){S}^{2} ,$$

which is comparable to the reported ($1+\mu {S}^{2})$ behavior for the low variation range^3^.

We obtain the following value for d in Eq. (25) by applying Chachiyo et al.’s suggested $\mu =8/27$:

26 $${d}^{2}+\frac{4\pi d}{3\left(2-\sqrt{\frac{2}{I}} \right)}=\frac{8}{27} \Rightarrow d=\frac{2}{9}\left(\sqrt{9{\pi }^{2}{\left(2-\sqrt{\frac{2}{I}} \right)}^{-2}+6}-3\pi {\left(2-\sqrt{\frac{2}{I}}  \right)}^{-1}\right).$$

We ultimately achieved the non-experimental exchange functional in this work by using the value of d found in Eq. (26) and defining the new variable $x=4\pi S/3$:

27 $$F\left(x\right)=\frac{\begin{array}{c}\left(\sqrt{9{\pi }^{2}{\left(2-\sqrt{\frac{2}{I}} \right)}^{-2}+6}-3\pi {\left(2-\sqrt{\frac{2}{I}} \right)}^{-1}\right){x}^{2}+({\frac{3}{4\pi }\left(\sqrt{9{\pi }^{2}{\left(2-\sqrt{\frac{2}{I}} \right)}^{-2}+6}-3\pi {\left(2-\sqrt{\frac{2}{I}} \right)}^{-1}\right)}^{2}{x}^{2}\\ +\left(\sqrt{9{\pi }^{2}{\left(2-\sqrt{\frac{2}{I}} \right)}^{-2}+6}-3\pi {\left(2-\sqrt{\frac{2}{I}} \right)}^{-1}\right)x+\frac{4\pi }{3})\left(2-\sqrt{\frac{2}{I}} \right)\left(L\left(-\alpha {\left(\frac{3}{4\pi }\right)}^{\frac{-3}{\sqrt{\frac{2}{I}} -2}}{x}^{\frac{-3}{\sqrt{\frac{2}{I}} -2}}\right)+1\right)\end{array}}{\left(\left(\sqrt{9{\pi }^{2}{\left(2-\sqrt{\frac{2}{I}} \right)}^{-2}+6}-3\pi {\left(2-\sqrt{\frac{2}{I}} \right)}^{-1}\right)x+\frac{4\pi }{3}\right)\left(2-\sqrt{\frac{2}{I}} \right)\left(L\left(-\alpha {\left(\frac{3}{4\pi }\right)}^{\frac{-3}{\sqrt{\frac{2}{I}} -2}}{x}^{\frac{-3}{\sqrt{\frac{2}{I}} -2}}\right)+1\right)}.$$

The exchange energy was calculated using this F(x) as follows:

28 $${E}_{x}=\int \rho {\varepsilon }_{x}\frac{\begin{array}{c}\left(\sqrt{9{\pi }^{2}{\left(2-\sqrt{\frac{2}{I}} \right)}^{-2}+6}-3\pi {\left(2-\sqrt{\frac{2}{I}} \right)}^{-1}\right){x}^{2}+({\frac{3}{4\pi }\left(\sqrt{9{\pi }^{2}{\left(2-\sqrt{\frac{2}{I}} \right)}^{-2}+6}-3\pi {\left(2-\sqrt{\frac{2}{I}} \right)}^{-1}\right)}^{2}{x}^{2}\\ +\left(\sqrt{9{\pi }^{2}{\left(2-\sqrt{\frac{2}{I}} \right)}^{-2}+6}-3\pi {\left(2-\sqrt{\frac{2}{I}} \right)}^{-1}\right)x+\frac{4\pi }{3})\left(2-\sqrt{\frac{2}{I}} \right)\left(L\left(-\alpha {\left(\frac{3}{4\pi }\right)}^{\frac{-3}{\sqrt{\frac{2}{I}} -2}}{x}^{\frac{-3}{\sqrt{\frac{2}{I}} -2}}\right)+1\right)\end{array}}{\left(\left(\sqrt{9{\pi }^{2}{\left(2-\sqrt{\frac{2}{I}} \right)}^{-2}+6}-3\pi {\left(2-\sqrt{\frac{2}{I}} \right)}^{-1}\right)x+\frac{4\pi }{3}\right)\left(2-\sqrt{\frac{2}{I}} \right)\left(L\left(-\alpha {\left(\frac{3}{4\pi }\right)}^{\frac{-3}{\sqrt{\frac{2}{I}} -2}}{x}^{\frac{-3}{\sqrt{\frac{2}{I}} -2}}\right)+1\right)}{d}^{3}r\hspace{0.17em}\hspace{0.17em}.$$

We combined this exchange energy with the subsequent correlation energy to create a new exchange–correlation energy for DFT computations:

29 $${E}_{c}=\int n{\varepsilon }_{c}{\left(1+{t}^{2}\right)}^{\frac{h}{{\varepsilon }_{c}}}{d}^{3}r .$$

We examine the accuracy of the introduced exchange–correlation energy for the set of atoms and molecules using Siam Quantum software and calculating Lambert function (Appendix A)^11,12^.

## Results and discussion

**Figure 2:**
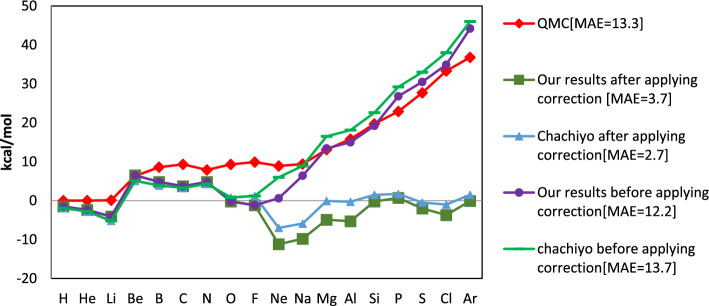
The mean absolute errors of the estimated total energies of single atoms. For atoms smaller than Ne, the fit performance is excellent; however, it is less precise for bigger atoms.

The suggested exchange energy and functional in Eqs. (28) and (29) offers a very accurate total energy for a set of the atoms and molecules in the first and second rows of the periodic table. Figure 2 illustrates the errors of the estimated total energies of single atoms. For atoms smaller than Ne, the fit performance is excellent; however, it is less precise for bigger atoms.

**Figure 3:**
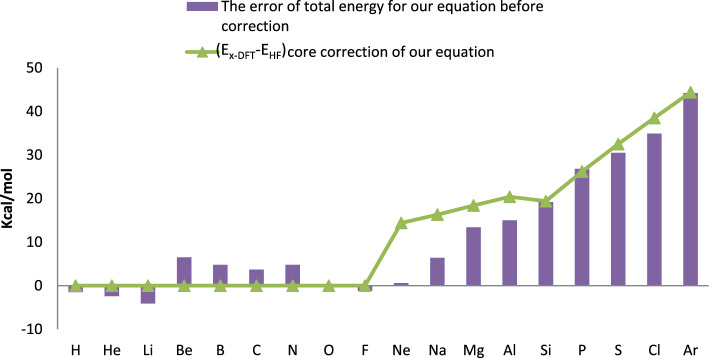
(Ex-DFT-EHF) core correction and the error of total energy for neutral atoms.

A quick correction approach utilized to increase the accuracy of total energy^1^. This approach is predicated on taking into account how core electrons affect the quantity of energy. Exchange functional performs exceptionally well for atoms smaller than Ne, but it performs less accurately for larger atoms. A precise and straightforward adjustment for atoms and molecules can be applied to overcome this issue. For each core ion, such as the magnesium ion, we first compute the exchange energy error using the Hartree–Fock computations. Hartree–Fock orbitals are the results of this. Next, we compute the DFT exchange energy (28) using density equation and the Hartree–Fock exchange energy utilizing these orbitals. As shown in Fig. 2, the errors were decreased after applying the correction. The MAE of the entire set of 56 molecules was only 3.7 kcal/mol. (E_xDFT_–E_HF_) for core electrons are nearly identical to the errors of the predicted total energies, as Fig. 3 illustrates. We take into account the 1S^2^ orbital as the core electrons from the H atom to the Al atom in our error correction technique, and the 2S^2^2P^6^ orbitals for the atoms larger than the Al atom (Fig. 3).

**Figure 4:**
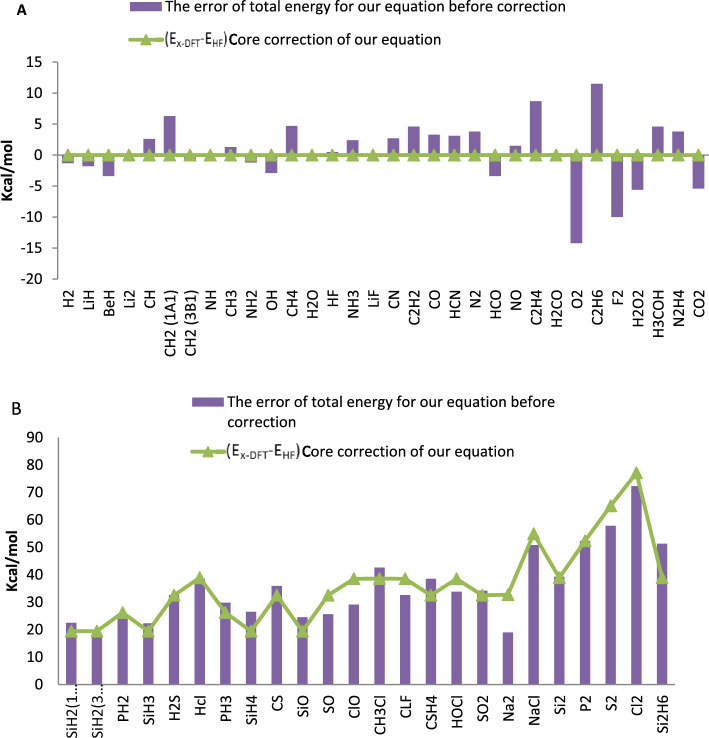
(Ex-DFT-EHF) core correction and the error of total energy for molecules including (**A**) first and second row atoms and (**B**) third row atoms.

In Fig. 4, this correction method depicted for molecules containing atoms from the first, second, and third rows of the periodic table.

For molecules containing atoms from the third row, this method very precisely illustrates the equality of the core electrons with the errors of predicted total energies.

**Figure 5:**
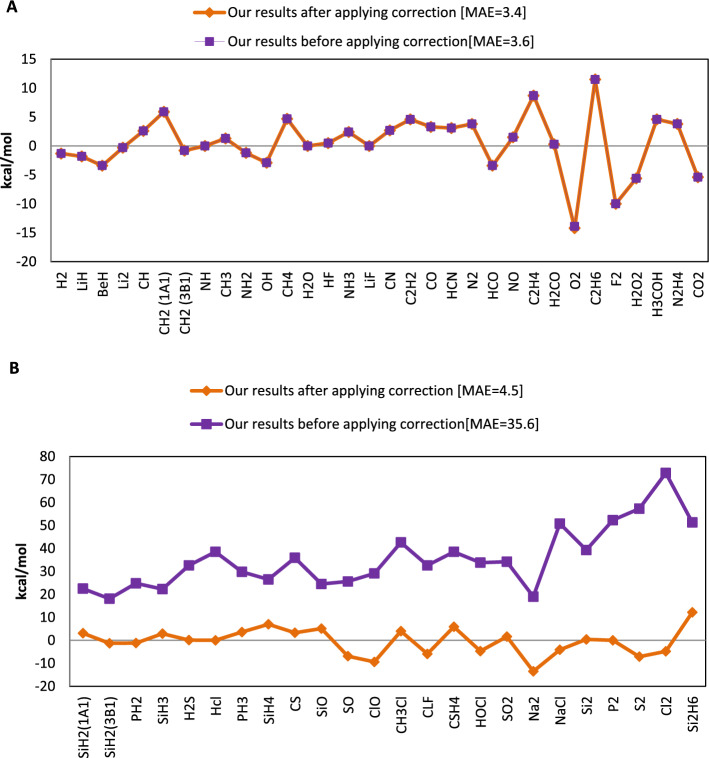
The errors of total energy obtained using our exchange functional in comparison to the experimental data for the total energy (kcal/mol) of molecules that (**A**) contain atoms from the first and second rows of the periodic table and (**B**) contain atoms from the third row of the periodic table.

The mean error (ME) of the total energy for the molecules containing the first, the second and the third rows of periodic table atoms is compared in Fig. 5. As shown in the caption of Fig. 5, the absolute mean error (MAE) for the first and second row molecules is better than the third row. These values for MAE are four times more accurate than the QMC result.

Tables 1 and 2 present a summary of the total energy produced by our developed XC functional as well as reference energies of atoms and molecules for computing the error value^13^.

**Table 1:** The total energy of examined atoms (versus Hartree)^13^.

Atoms	Total energy (experimental)	Total energy (our model)
H	− 0.5000	− 0.502554
He	− 2.9037	− 2.907579
Li	− 7.4781	− 7.484782
Be	− 14.6674	− 14.65707
B	− 24.6539	− 24.64627
C	− 37.845	− 37.83908
N	− 54.5892	− 54.58151
O	− 75.0673	− 75.06775
F	− 99.7339	− 99.73586
Ne	− 128.9376	− 128.95558
Na	− 162.2546	− 162.27037
Mg	− 200.053	− 200.06092
Al	− 242.346	− 242.35457
Si	− 289.359	− 289.35931
P	− 341.259	− 341.25789
S	− 398.11	− 398.10728
Cl	− 460.148	− 460.15389
Ar	− 527.54	− 527.54032

**Table 2:** The total energy of examined molecules (versus Hartree)^13^.

Molecules	Total energy (experimental)	Total energy (our model)
Li_2_	− 14.9951	− 14.99553
CH	− 38.4788	− 38.47462
CH_2_ (1A1)	− 39.1346	− 39.12451
CH_2_ (3B1)	− 39.1484	− 39.14978
NH	− 55.2227	− 55.22271
CH_3_	− 39.8355	− 39.83338
NH_2_	− 55.8794	− 55.88145
OH	− 75.7371	− 75.74185
CH_4_	− 40.5158	− 40.50829
H_2_O	− 76.4383	− 76.43823
HF	− 100.459	− 100.4600
H_2_	− 1.1745	− 1.176598
LiH	− 8.0704	− 8.073238
CO	− 113.326	− 113.3207
HCN	− 93.4311	− 93.42615
N_2_	− 109.542	− 109.5360
HCO	− 113.857	− 113.8624
NO	− 129.905	− 129.9022
C_2_H_4_	− 78.5888	− 78.57484
H_2_CO	− 114.509	− 114.5084
O_2_	− 150.327	− 150.3493
C_2_H_6_	− 79.8274	− 79.80910
F_2_	− 199.53	− 199.5463
H_2_O_2_	− 151.564	− 151.5724
H_3_COH	− 115.731	− 115.7231
N_2_H_4_	− 111.878	− 111.8715
CO_2_	− 188.601	− 188.6098
BeH	− 15.2468	− 15.25018
SiH_2_(3B1)	− 290.569	− 290.5712
H_2_S	− 399.403	− 399.4023
Hcl	− 460.819	− 460.8197
PH_3_	− 343.146	− 343.1403
SiH_4_	− 291.874	− 291.8631
CS	− 436.229	− 436.2236
SiO	− 364.734	− 364.7255
SO	− 473.378	− 473.3893
ClO	− 535.32	− 535.3352
CH_3_Cl	− 500.124	− 500.1178
CLF	− 559.982	− 559.9915
CSH_4_	− 438.712	− 438.7029
HOCl	− 535.98	− 535.9870
SO_2_	− 548.659	− 548.6561
Na_2_	− 324.536	− 324.5576
LiF	− 107.434	− 107.4343
SiH_2_ (1A1)	− 290.602	− 290.5969
Cl_2_	− 920.39	− 920.3977
Si_2_H_6_	− 582.567	− 582.5472
NaCl	− 622.561	− 622.5670
Si_2_	− 578.839	− 578.8378
P_2_	− 682.704	− 682.7042
S_2_	− 796.384	− 796.3956
PH_2_	− 342.504	− 342.5055
SiH_3_	− 291.221	− 291.2170
CN	− 92.725	− 92.72058
C_2_H_2_	− 77.3355	− 77.32817
NH_3_	− 56.5647	− 56.56075

The ME and MAE for neutral atoms obtained with our functional are less than CM, as Table 3 illustrates. Our exchange functional’s ME is nearly zero for the total energy of the 56 molecules after rapplying correction, making it more accurate than the CM’s reported 1 kcal/mol value, even though it yielded a 3.9 kcal/mol MAE, which is higher than the CM’s reported 3.5 kcal/mol value. Table 4 illustrates the errors of the total energy for our model and the CM.

**Table 3:** The comparison of the our and Chachiyo models total energy errors for neutral atoms.

Total energy	Chachiyo model	Chachiyo model with correction	Our model	Our model with correction
ME (for all neutral atoms)	12.6	− 0.1	11.2	− 1.4
MAE (for all neutral atoms)	13.7	2.7	12.2	3.7
ME (for first and second row neutral atoms)	1.5	0.2	1.1	− 0.1
MAE (for first and second row neutral atoms)	3.4	3.5	3.0	4.0

The unit of every energy is kcal/mol.^1^.

**Table 4:** The comparison of the our and Chachiyo models total energy errors for examined 56 molecules.

Total energy	Chachiyo model	Chachiyo model with correction	Our model	Our model with correction
ME (for 56 molecules)	16.9	1.0	15.5	0.0
MAE (for 56 molecules)	18.5	3.5	17.3	3.9
ME (for molecules made up from first and second row atoms)	0.4	0.4	0.5	0.4
MAE (for molecules made up from first and second row atoms)	3.2	3.2	3.6	3.4

The unit of every energy is kcal/mol.^1^.

**Figure 6:**
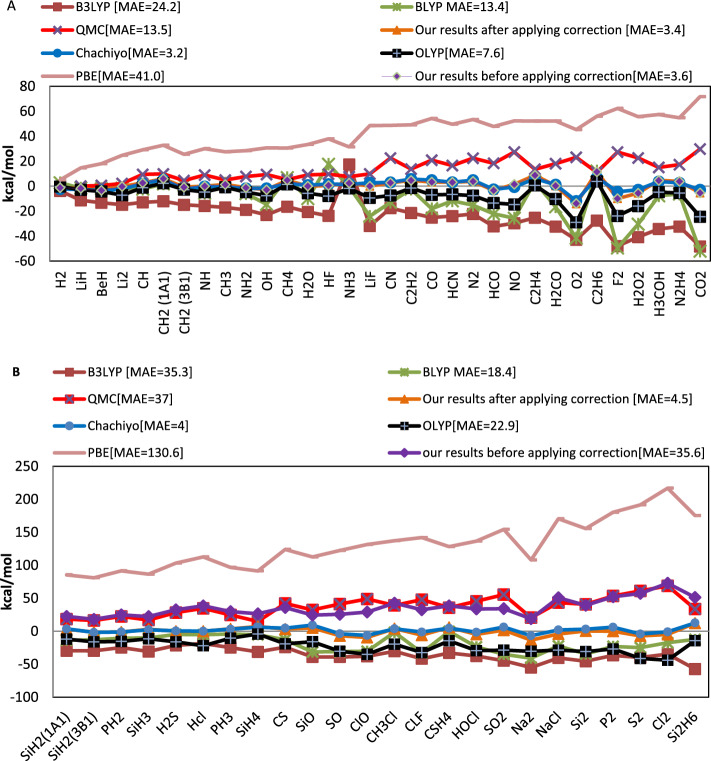
The total energy mean absolute error (kcal/mol) of various DFT approaches compared to the experimental values for molecules that contain (**A**) atoms from the first and second rows of the periodic table and (**B**) atoms from the third row of the periodic table.

Additionally, we compared our total energy’s ME with the results of the well-known B3LYP, BLYP, PBE, OLYP, QMC, and CE methods^1,14–16^. The ME for the reference energies and for the molecules of the first and second rows of the periodic table is approximately 0.4 kcal/mol and which is same as our functional result as illustrated in Fig. 6^12^. Our functional estimates 17.3 kcal/mol MAE for the total energy without applying any corrections. For the CM, this error was equal to 18.5 kcal/mol.

Although the errors are only a few kcal/mol for molecules made up of atoms from the first and second rows of the periodic table, they drastically increase for molecules composed of toms from the third row. This increase in errors is caused by the core electrons.

**Figure 7:**
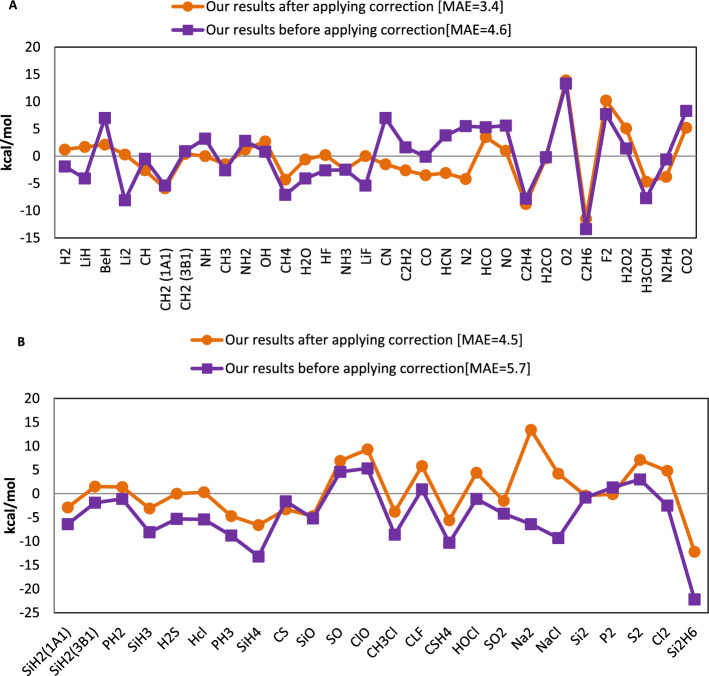
The bond energies mean absolute error (kcal/mol) obtained by our new functional compared to the experimental value for (**A**) molecules containing atoms of the first and second rows of the periodic table and (**B**) molecules containing atoms of the third row of the periodic table.

Additionally, we used both corrected and uncorrected energies to evaluate molecules bond energy (E_b_) using^1^:

30 $${E}_{b}={\sum }_{A\in atoms}{E}_{total}^{(A)}-{E}_{total}^{(M)}\text{\hspace{0.17em}\hspace{0.17em},}$$

where *E*^*(A)*^ and *E*^*(M)*^ represent the total energies of atoms and molecules, respectively. In Fig. 7, the bond energy errors are displayed. MAE for molecules having atoms from the first and second rows of periodic table, from the third row of periodic table, and for all 58 molecules is equal to 4.6, 5.7 and 5.1, respectively. The MAE value for all molecules is decreased to 0.0 kcal/mol considering the core correction in Eq. (30). In this case, the atomic energies from reference^13^, along with the corrected molecule energy are employed.

**Figure 8:**
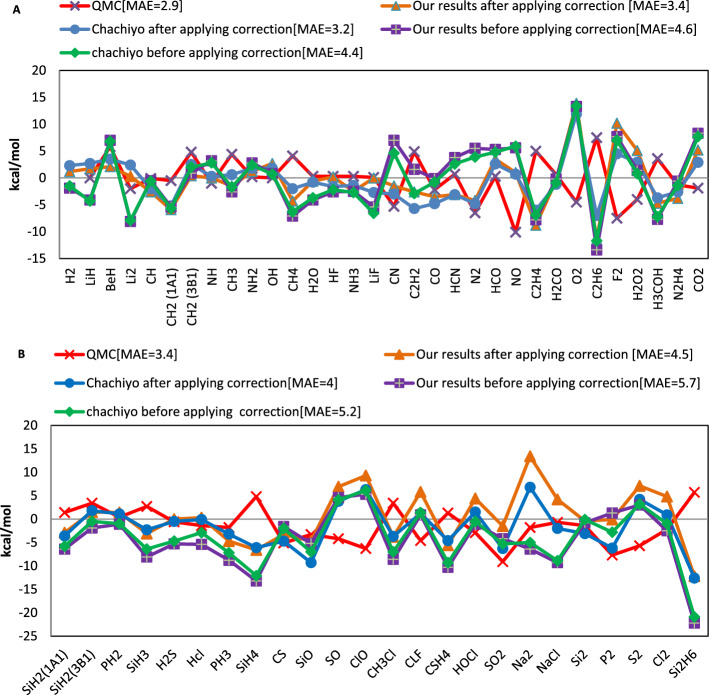
The bond energies mean absolute error (kcal/mol) obtained by various models compared to the experimental values for (**A**) molecules containing atoms of the first and second rows of the periodic table and (**B**) molecules containing atoms of the third row of the periodic table.

In Fig. 8, the MAE for our bond energy is compared with the QMC and CM approaches.

Table 5 compares the bond energy errors for our model and the CM.

**Table 5:** The comparison of the bond energy errors for our and Chachiyo models.

Bond energy error	Chachiyo model	Chachiyo model with correction	Our model	Our model with correction
ME (for 56 molecules)	− 1.9	− 1.0	− 1.9	3.9
MAE (for 56 molecules)	4.7	3.5	5.1	0.0
ME (for molecules made up from first and second row atoms)	− 0.3	− 0.4	0.0	− 0.4
MAE (for molecules made up from first and second row atoms)	4.4	3.2	4.6	3.4

The unit of every energy is kcal/mol.

Dipole moments are also analyzed using our employed electron density at this work. Our results and experimental data for dipole moments are summarized in Table 6.

**Table 6:** The experimental and our results for zero point energy and dipole moment of molecules.

Molecules	E_ZP_			Dipole moments		
	Experimental	Our calc (kc/mol)	ME	Experimental	Our calc (Debye)	ME
H_2_	6.2	6.4	0.2	0.0	0.0	0.0
LiH	2.0	2.1	0.1	5.87	5.74	− 0.13
BeH	2.9	3.0	0.1	0.25	0.25	0.0
Li_2_	0.5	0.5	0.0	0.0	0.0	0.0
CH	4.0	4.1	0.1	1.45	1.42	− 0.03
NH	4.6	4.6	0.0	1.39	1.49	0.1
NH_2_	11.5	12.0	0.5	1.76	1.76	0.0
OH	5.3	5.5	0.2	1.65	1.62	− 0.03
CH_4_	27.1	27.2	0.1	0.0	0.0	0.0
H_2_O	12.9	13.1	0.2	1.85	1.85	0.0
HF	5.9	6.0	0.1	1.82	1.77	− 0.05
NH_3_	20.6	21.0	0.4	1.47	1.50	0.03
LiF	1.3	1.4	0.1	6.31	6.13	− 0.18
CN	2.9	3.1	0.2	1.14	1.14	0.0
C_2_H_2_	15.3	15.3	0.0	0.0	0.0	0.0
CO	3.1	3.1	0.0	0.11	0.19	0.08
HCN	8.7	9.0	0.3	2.98	2.90	− 0.08
N_2_	3.4	3.6	0.2	0.0	0.0	0.0
HCO	7.8	7.9	0.1	1.39	1.39	0.0
NO	2.7	2.9	0.2	0.16	0.21	0.05
H_2_CO	16.1	16.1	0.0	2.32	2.19	− 0.13
O_2_	2.3	2.4	0.1	0.0	0.0	0.0
F_2_	1.3	1.4	0.1	0.0	0.0	0.0
H_2_O_2_	−	16.3	−	1.57	1.72	0.15
CH_4_O	−	31.7	−	1.69	1.56	− 0.13
N_2_H_4_	−	32.6	−	1.75	1.87	0.12
CO_2_	6.2	6.3	0.1	0.0	0.0	0.0
SiH_2_	7.2	7.4	0.2	0.26	0.26	0.0
H_2_S	9.2	9.2	0.0	0.98	1.05	0.07
HCl	4.2	4.3	0.1	1.11	1.13	0.02
PH_3_	14.6	14.7	0.1	0.57	0.70	0.13
SiH_4_	19.2	19.2	0.0	0.0	0.0	0.0
CS	1.8	1.9	0.1	1.95	1.93	− 0.02
SiO	1.8	1.8	0.0	3.09	2.83	− 0.26
SO	1.6	1.7	0.1	1.54	1.36	− 0.18
ClO	1.2	1.3	0.1	1.3	1.38	0.08
CH_3_Cl	−	23.3	−	1.88	1.87	− 0.01
ClF	1.1	1.2	0.1	0.88	0.75	− 0.13
CSH_4_	−	28.5	−	1.52	1.56	0.04
HOCl	8.0	8.1	0.1	1.40	1.53	0.13
SO_2_	4.3	4.6	0.3	1.62	1.48	− 0.14
Na_2_	0.2	0.2	0.0	0.0	0.0	0.0
NaCl	0.5	0.5	0.0	8.99	8.8	− 0.19
Si_2_	0.7	0.7	0.0	0.0	0.0	0.0
P_2_	1.1	1.1	0.0	1.1	0.0	0.0
S_2_	1.0	1.1	0.1	1.0	0.0	0.0
Cl_2_	0.8	0.8	0.0	0.8	0.0	0.0
MAE			0.11	MAE		0.09
ME			0.11	ME		− 0.02

The units are in Debye^5^.

As summarized in Table 7, MAE is obtained 0.09 Debye which is comparable to the DFT-based estimations and better than CM^17^.

**Table 7:** The comparison of the dipole moment errors for our and Chachiyo models.

Dipole moment	Chachiyo model	Chachiyo model with correction	Our model	Our model with correction
ME	− 0.03		− 0.02	
MAE	0.11		0.09	

The unit of every energy is kcal/mol.^1^.

In contrast to classical mechanics, quantum systems fluctuate around zero-point energy (E_ZP_) even at absolute zero temperature. In order to calculate vibration frequencies, the accuracy of E_ZP_ should be increased. E_ZP_ can be calculated using Eq. (31) as^18^:

31 $${E}_{ZP}=\frac{1}{2}\sum h{\nu }_{i} ,$$

where i represent the frequency of a certain molecule and h is the Planck constant. Table 8 provides an overview of our findings and the experimental data for E_ZP_.

**Table 8:** The comparison of the zero point energy errors for our and Chachiyo models.

Zero point energy	Chachiyo model	Chachiyo model with correction	Our model	Our model with correction
ME	− 0.005		0.11	
MAE	0.12		0.11	

The unit of every energy is kcal/mol.^1^.

The MAE of the E_ZP_, as shown in Table 8, is 0.11 kcal/mol, which is comparable to the Chachiyo prediction^1^. In this investigation, the QZP-g basis set was employed.

We compared the errors of zero point energy for our and CM in Table 8.

As a result, in addition to the total energies, our exchange correlation can also reliably predict the dipole moment, E_B_ and E_ZP_.

We were curious to see how our exchange functional performed with various organic and solid substances. We also achieved satisfactory results for their dipole moment, zero-point energy, and total energy, as Table 9 shows.

**Table 9:** Comparison of three organic and six solid materials’ zero point energy (E_ZP_), dipole moment, and total energy^19–23^.

Molecules	E_ZP_ (kcal/mol)	Experimental E_ZP_ (kcal/mol)	E_ZP_ error	Dipole moment (Debye)	Experimental dipole moment	Dipole moment error	Total energy (Hartree)	Experimental total energy (Hartree)	Total energy error (Kcal/mol)
Thiadiazole (1-3-4 C_2_H_2_N_2_S)	26.80	27.4 [22]	0.6	2.76	1.57 [23]	1.19	− 584.99	− 584.92 [22]	− 43.8
Thiophene (C_4_H_4_S)	43.90	43.6 [22]	0.3	0.71	0.55 [19, 20]	0.16	− 552.92	− 552.95 [22]	− 20.33
Benzothiazole (C_7_H_5_NS)	65.34	−	−	1.39	1.46 [19, 20]	0.07	− 714.05	−	−
BeO	2.05	2.05 [22]	0.0	5.31	−	−	− 89.849	− 89.846 [22]	1.96

Dashed line shows that experimental value is not available.

Siam-Quantum can be used to compute additional variables, such as molecular vibrations and the related normal modes and forces, which were computed for solids. Table 10 presents an overview of the results achieved for these parameters.

**Table 10:** Molecular vibration properties of some solid materials’.

Material	Atoms	Vibration wave number (Cm^–1^)	Normal mode vibration			Forces (Hartrees/Bohr)		
						F_x_	F_y_	F_z_
BeO	Be	1440.6	0.00	0.00	− 0.2669	0.00000	− 0.00000	− 0.002106
	O		− 0.00	0.00	0.1496	− 0.00000	0.000000	0.004986
LiSi	Li	379.0	0.0684	− 0.0667	− 0.3230	0.00009	− 0.000138	− 0.000331
	Si		− 0.0171	0.0165	0.0819	− 0.00006	0.000212	0.000075
Na_2_S	Na	301.7	0.0658	0.0659	0.0661	0.000585	0.000584	0.000577
	S		− 0.0919	− 0.0919	− 0.0920	0.000099	0.000099	0.000099
BeCl_2_	Be	844.5	0.0414	0.0414	− 0.0492	0.005446	0.005452	− 0.006449
	Cl		− 0.1606	− 0.1609	0.1913	− 0.003795	− 0.003800	0.004498
MgF_2_	Mg	709.3	− 0.1209	− 0.1209	0.0000	0.002517	0.002517	0.000007
	F		0.0963	0.0963	− 0.0000	0.000928	0.000928	0.000007
MgB_2_	Mg	351.1	0.0000	0.0812	0.0803	− 0.00000	0.000065	0.000068
	B		0.0000	− 0.1776	− 0.1757	0.000000	0.000617	0.000609
LiH	Li	333.5	0.0358	0.0716	− 0.1074	0.014346	0.014346	0.014316
	H		− 0.2490	− 0.4978	0.7467	− 0.014342	− 0.014342	− 0.014312
BeH	Be	2270.6	0.0000	− 0.0000	0.1056	0.000000	0.000000	− 0.004285
	H		0.0000	− 0.0000	− 0.9447	− 0.000000	0.000000	0.004291
Li_2_	Li	391.1	− 0.0000	0.0000	− 0.2670	0.000000	0.000000	0.002038
	Li		− 0.0000	− 0.0000	− 0.2670	0.000000	0.000000	− 0.002038
LiF	LI	845	0.0000	− 0.0000	− 0.3229	0.000000	0.000000	− 0.030607
	F		− 0.0000	− 0.0000	0.1189	− 0.000000	− 0.000000	0.031051
Na_2_	Na	144.6	0.0258	− 0.1452	− 0.0000	− 0.000000	− 0.000000	0.054220
	Na		− 0.0258	0.1452	0.0000	0.000000	− 0.000000	− 0.054220
NaCl	Na	410.7	0.0000	− 0.0000	0.1621	− 0.00000	0.00000	0.026416
	Cl		− 0.0000	0.0000	− 0.1064	0.000000	− 0.00000	− 0.026457
SiO	Si	1370.4	0.0000	− 0.0000	0.1140	− 0.00000	− 0.00000	0.049936
	O		− 0.000	0.0000	− 0.1994	0.000000	0.000000	− 0.049961

## Conclusion

In this paper, we derive an accurate and straightforward exchange functional that can be applied in the intermediate, slow and fast density variation limits. According to calculations on first, second and third rows of periodic table neutral atoms and 56 molecules, our exchange functional accurately predicts the total energy, dipole moment, bond and zero point energies. The total energy mean error of our functional is 0.0 for examined 56 molecules which shows that it concludes more accurate results than other exchange functionals. The mean absolute error of the total energy for the mentioned molecules are calculated was obtained 3.9 kcal/mol which is higher than the result of CM calculations but is better than the QMC result. The error of our functional for the third row of the periodic table's atoms was greater than that of the first and second rows’ atoms because of the role of core atoms. Our functional results for neutral atoms are comparable to CM as evidenced by its low ME and MAE. The MAE for the estimated zero-point energy and dipole moments also confirms the accuracy of our new exchange functional.

### Supplementary Information

Supplementary Information.

## Data Availability

All data generated or analyzed during this study are included in this published article. If required, any data are available from the corresponding author on reasonable request.
