# Supplementary material for: Introducing a new exchange functional by altering the electron density’s ionization dependency in density functional theory
Source: Sci Rep. 2024 Feb 8;14:3226. doi: 10.1038/s41598-024-53341-4 (PMC10853517; doi:10.1038/s41598-024-53341-4)
Supplement: Supplementary file 1 — Supplementary Information. [file 41598_2024_53341_MOESM1_ESM.docx]

Appendix A

W(1)= 0.5671432904097838730

W(2)= 0.8526055020137254914

W(20)=2.2050032780240599705

#include <math.h>

#include <stdio.h>

double LambertW(const double z);

const int dbgW=0;

double LambertW(const double z) {

int i;

const double eps=4.0e-16, em1=0.3678794411714423215955237701614608;

double p,e,t,w;

if (dbgW) fprintf(stderr,"LambertW: z=%g\n",z);

if (z<-em1 || isinf(z) || isnan(z)) {

fprintf(stderr,"LambertW: bad argument %g, exiting.\n",z); exit(1);

}

if (0.0==z) return 0.0;

if (z<-em1+1e-4) { // series near -em1 in sqrt(q)

double q=z+em1,r=sqrt(q),q2=q*q,q3=q2*q;

return

-1.0

+2.331643981597124203363536062168*r

-1.812187885639363490240191647568*q

+1.936631114492359755363277457668*r*q

-2.353551201881614516821543561516*q2

+3.066858901050631912893148922704*r*q2

-4.175335600258177138854984177460*q3

+5.858023729874774148815053846119*r*q3

-8.401032217523977370984161688514*q3*q; // error approx 1e-16

}

/* initial approx for iteration... */

if (z<1.0) { /* series near 0 */

p=sqrt(2.0*(2.7182818284590452353602874713526625*z+1.0));

w=-1.0+p*(1.0+p*(-0.333333333333333333333+p*0.152777777777777777777777));

} else

w=log(z); /* asymptotic */

if (z>3.0) w-=log(w); /* useful? */

for (i=0; i<10; i++) { /* Halley iteration */

e=exp(w);

t=w*e-z;

p=w+1.0;

t/=e*p-0.5*(p+1.0)*t/p;

w-=t;

if (fabs(t)<eps*(1.0+fabs(w))) return w; /* rel-abs error */

}

/* should never get here */

fprintf(stderr,"LambertW: No convergence at z=%g, exiting.\n",z);

exit(1);

}

#ifdef TESTW

/* test program... */

int main() {

int i;

double z,w,err;

for (i=0; i<100; i++) {

z=i/100.0-0.3678794411714423215955; w=LambertW(z);

err=exp(w)-z/w;

printf("W(%8.4f)=%22.16f, check: exp(W(z))-z/W(z)=%e\n",z,w,err);

}

for (i=0; i<100; i++) {

z=i/1.0e-1-0.3; w=LambertW(z);

err=exp(w)-z/w;

printf("W(%8.4f)=%22.16f, check: exp(W(z))-z/W(z)=%e\n",z,w,err);

}

return 0;

}

#endif

#ifdef INTW

int main() {

int i,n=1000;

double w,z,s=0,err;

for (i=1; i<=n; i++) {

z=i/(double)n;

w=LambertW(1/z)/(1+z);

s+=w;

printf("%8.4f %8.4f\n",z,w);

}

fprintf(stderr,"%8.4f\n",exp(s/n/log(2)));

return 0;

}

#endif
